# Are caregivers for older adults satisfied with the care robots in the workplace? A cross-sectional study in China

**DOI:** 10.3389/fpubh.2026.1788463

**Published:** 2026-04-29

**Authors:** Tingting Yue, Ying Wang, Ziqiong Liu

**Affiliations:** 1 School of Economics and Management, Anhui University of Chinese Medicine, Hefei, Anhui, China; 2 Key Laboratory of Data Science and Innovative Development of Chinese Medicine in Anhui Province,Philosophy and Social Sciences, Hefei, Anhui, China; 3 School of Management, Hefei University of Technology, Hefei, Anhui, China

**Keywords:** AI self-efficacy, care robot, caregivers for older adults, job satisfaction, product interaction perception, usage satisfaction

## Abstract

**Background:**

With the aim of enhancing the quality of older adult assistance services and optimizing service processes, the Chinese government has actively promoted a technology empowerment strategy, selecting pilot cities to conduct application demonstrations of care robots. However, significant individual differences exist in the acceptance of care robots among caregivers for older adults, highlighting the need for systematic empirical research to evaluate the practical feasibility of care robots in older adults care scenarios.

**Purpose:**

This study examines the motivations of caregivers for older adults toward care robots during caregiving processes, aiming to advance innovation in information technology and public healthcare.

**Methods:**

This study constructs an analytical framework to explore the usage satisfaction of caregivers for older adults with care robots and their influencing factors. A total of 544 caregivers for older adults from 15 older adult care institutions were enrolled. Data were analyzed using SPSS and SmartPLS software.

**Results:**

Product interaction perception of care robots has a notably affirmative influence on usage satisfaction. Job satisfaction (*β* = 0.328, *p* < 0.001) and AI self-efficacy (*β* = 0.171, *p* < 0.001) have a marked affirmative influence on usage satisfaction. The research shows that product interaction perception and AI self-efficacy have a prominent influence on the usage satisfaction of care robots. Additionally, caregivers for older adults with higher job satisfaction exhibit greater usage satisfaction.

**Conclusion:**

The purpose of this study is to investigate caregivers’ satisfaction with the use of care robots. The findings provide additional crucial evidence that product interaction perception and AI self-efficacy are associated with the usage satisfaction of caregivers for older adults.

## 1 Introduction

Globally, as societies continue to age and the demand for healthcare services grows significantly, the supply of new nursing professionals remains insufficient (1). This has led to a prominent nursing shortage issue in public health. In response to this crisis, scholars are increasingly leveraging artificial intelligence (AI) and big data to design and develop nursing robots (2). The goal is to alleviate the burden on nurses, enabling them to focus on more complex, specialized clinical tasks. Countries around the world are confronted with the severe challenge of population aging (3). In China, it is projected that the country will enter a stage of severe aging by 2035 (4). This rapid demographic transition has resulted in a substantial increase in the need for older adult care services. However, the current older adult care industry faces a critical shortage of professional caregivers (5). In response, the Chinese government has promoted smart aging, leveraging AI technologies to address these challenges and facilitate the transition between nursing institutions and older adult care organizations (6). To this end, the government of China (GOC) has chosen some regions with robust AI infrastructure to conduct pilot projects on AI applications in social older adults care, while promoting the deployment of care robots in older adults care institutions.

As primary service providers within the older adults care sector, professional caregivers assume a diverse array of responsibilities, encompassing physical assistance, emotional support, and rehabilitation care (7). The inherent nature of their work entails high-intensity manual labor, such as assisting disabled seniors with activities of daily living, and sustained emotional engagement, including the provision of psychological comfort (8). These demands, compounded by challenging working conditions and limited societal recognition, contribute to low job satisfaction, elevated burnout rates, and substantial staff turnover, ultimately compromising the overall quality of older adults care services (9). Care robots are a category of intelligent service robots primarily designed to monitor, accompany, and provide care for individuals with unique needs, assisting humans in performing specific functions or fulfilling certain requirements (10). They represent a comprehensive technological solution aimed at addressing the shortage of care personnel in an aging society while enhancing care quality and the quality of life for the older adults through intelligent technologies (11). Currently, AI care robots are increasingly being deployed in childcare, eldercare, psychological therapy, and other healthcare applications.

With the advancement of digital technologies, technological empowerment has emerged as a promising strategy to enhance both operational efficiency and employee wellbeing (12). Older adults care institutions have increasingly adopted advanced tools such as care robots to assist with caregiving tasks. These robotic systems encompass various types, ranging from daily living assistants and medical support units to social companions, and offer functionalities including health monitoring, mobility assistance, hygiene management, and interactive companionship (13). Capable of providing round-the-clock (24/7) services, they help reduce human error, alleviate the physical burden on older adult caregivers, streamline service delivery, and improve overall efficiency (14, 15). Nevertheless, existing studies indicate mixed perceptions among healthcare professionals, including nurses and midwives, regarding the utility of social assistance robots in health and social care settings (16). Some caregivers for older adults even express skepticism or dissatisfaction with robotic integration (17). Therefore, further empirical investigation is necessary to validate the feasibility of deploying care robots within eldercare environments. Understanding the mechanisms influencing usage satisfaction of caregivers for older adults with care robots holds significant practical value for policy formulation and service innovation.

The UTAUT model (Unified Theory of Acceptance and Use of Technology) has been widely used to understand individual behavior related to technology adoption (18). It emphasizes that users’ perceived utility of a technological product constitutes the foundation for its acceptance (19). However, UTAUT provides limited insight into how these cognitive perceptions evolve into emotional-level satisfaction, a critical mechanism in determining long-term technology usage (20, 21). Furthermore, AI self-efficacy, which reflects an individual’s confidence in interacting with AI systems, exerts a remarkable effect on the user experience of caregivers for older adults (22–24). Nevertheless, this dimension has been insufficiently addressed within the original UTAUT model. Therefore, this study extends the UTAUT framework by integrating AI self-efficacy and proposes a theoretical model to examine older adult caregivers’ usage satisfaction with care robots, thereby broadening the theoretical applicability of the UTAUT model to geriatric care.

Satisfaction theory provides a conceptual foundation for analyzing individuals’ subjective evaluations and affective responses toward products or services (25–27). Within the context of older adults care, when caregivers for older adults compare their actual experiences with care robots against their initial expectations, emotional reactions are elicited (28). These reactions subsequently shape their attitudes toward continued use. However, existing studies that integrate UTAUT and satisfaction theory have largely overlooked the mediating role of job satisfaction, leading to an incomplete understanding of how usage satisfaction with care robots is formed in human–robot collaborative environments.

To address these gaps, this study integrates UTAUT with satisfaction theory by incorporating key constructs such as product interaction perception, AI self-efficacy, and job satisfaction into a unified analytical framework. This approach aims to explore the mechanisms influencing usage satisfaction of caregivers of older adults with care robots. The proposed model not only extends the applicability of UTAUT to the domain of older adult healthcare but also contributes a novel theoretical lens for examining the formation of technology usage satisfaction, offering both academic and practical implications.

## 2 Hypotheses development

### 2.1 Product interaction perception and usage satisfaction

The UTAUT model puts forward the idea that there are four key constructs, namely performance expectancy, effort expectancy, social influence, and facilitating conditions, which impact users’ behavioral intention as well as their actual usage behavior regarding technology (29). This model has been widely applied across diverse domains, including technology adoption, user satisfaction analysis, continuance usage research, and technology recommendation studies (30–33). Usage satisfaction is recognized as a critical indicator for evaluating the successful implementation of information systems (34).

Within the context of older adults care institutions, care workers’ usage satisfaction with care robots is largely shaped by their perceptions of multiple factors during the usage process. Given its explanatory power, UTAUT has been frequently adopted in usage satisfaction studies. For instance, Joshi identified that facilitating conditions significantly influence usage satisfaction with chatbots (35). Kim applied the UTAUT framework to examine healthcare professionals’ satisfaction with AI-based medical devices, revealing that performance expectancy and effort expectancy are important indicators of user satisfaction (20). Similarly, Lee demonstrated that healthcare professionals’ performance expectancy and effort expectancy of social assistive robots can significantly influence their satisfaction with using them (36). Jung’s research found that when restaurants use service robots, customers’ performance expectancy and effort expectancy toward service robots have a significant positive impact on customers’ usage satisfaction (37).

In this study, performance expectancy refers to the extent to which caregivers for older adults believe that using care robots can help them perform better in their work (38). Effort expectancy in this study refers to the ease with which caregivers for older adults can use care robots (39). Facilitating conditions refer to the supportive factors available to caregivers for older adults when using care robots (40). Building on these findings, the present study incorporates performance expectancy, effort expectancy, and facilitating conditions into the dimension of product interaction perception. It is proposed that eolder adults caregivers’ product interaction perception with care robot in older adults care institutions will significantly influence their overall usage satisfaction. Based on the above theoretical foundation and empirical evidence, the subsequent hypotheses are formulated.

*H1a:* Performance expectancy has a significant positive effect on the usage satisfaction of caregivers for older adults with care robots.

*H1b:* Effort expectancy has a significant positive effect on the usage of caregivers for older adults with care robots.

*H1c:* Facilitating conditions have a significant positive effect on the usage satisfaction of caregivers for older adults with care robots.

### 2.2 Product interaction perception and job satisfaction

Job satisfaction refers to the positive emotional response that employees develop toward their work or professional experiences (41). It is recognized as a crucial psychological indicator in enterprise management for evaluating workforce wellbeing and organizational effectiveness (42). This construct may be influenced by both intrinsic psychological factors and extrinsic job-related conditions, and extensive research has been conducted globally to explore its antecedents and outcomes.

In the context of information technology or related product usage, empirical studies have demonstrated significant associations between system adoption and user job satisfaction (43). For instance, Voinea’s research indicated that when caregivers use robots to assist them in providing older adults care, their job satisfaction increases as their performance expectancy for robots increases (44). Zhang found that the implementation of intelligent medical information systems positively influences physicians’ job satisfaction (45). Kim applied the UTAUT framework to investigate healthcare professionals’ satisfaction with AI-based medical devices and identified performance expectancy and effort expectancy as key predictors of job satisfaction (20). Similarly, Maillet revealed that performance expectancy, effort expectancy, and facilitating conditions significantly enhanced nurses’ satisfaction with hospital ERP systems (46). In the context of this study, the attitudes of caregivers for older adults toward care robots within older adults care institutions significantly influence their overall job satisfaction levels (10). When caregivers for older adults believe that robots can effectively improve their work performance, they will develop a value perception, which in turn increases job satisfaction (47, 48). When caregivers for older adults perceive that robots are easy to learn and use, it can reduce technical anxiety, enhance self-efficacy, and thus improve satisfaction (36). Moreover, when caregivers for older adults perceive that there are more facilitating conditions for using robots, they will interpret this as a sense of support, thereby significantly improving job satisfaction (49).

Therefore, the present study posits that older adults caregivers’ perceived experiences with care robots in institutional settings significantly influence their job satisfaction. Based on the above theoretical and empirical foundations, the following hypotheses are proposed.

*H2a:* Performance expectancy has a significant positive effect on the job satisfaction of caregivers for older adults.

*H2b:* Effort expectancy has a significant positive effect on the job satisfaction of caregivers for older adults.

*H2c:* Facilitating conditions have a significant positive impact on the job satisfaction of caregivers for older adults.

### 2.3 AI self-efficacy, job satisfaction, and usage satisfaction

Self-efficacy refers to an individual’s belief in their ability to successfully carry out specific tasks or behaviors (50). It plays a key role in shaping motivation, behavioral persistence, and decision-making processes (51). Previous studies have indicated that within the context of information systems or technology use, AI self-efficacy reflects an individual’s understanding of their competence in utilizing next-generation digital tools to accomplish work-related tasks. Those who possess high self-efficacy are more inclined to persevere and make efforts when facing challenges (52), which can lead to improved job performance and adaptive work strategies, ultimately enhancing their satisfaction in professional environments (53).

Building on this concept, AI self-efficacy has emerged as an extended construct that describes individuals’ confidence in using AI systems or advanced digital technologies (54, 55). Berrezueta’s research indicated that incorporating AI tools, such as robots, into the treatment of attention-deficit/hyperactivity disorder in children can reduce the workload of healthcare providers, enhance their self-efficacy, and consequently have a positive impact on their job satisfaction (56).

In the context of older adults care institutions, care robots can take on some of the repetitive and tedious tasks involved in caregiving (10, 57). Caregivers for older adults with a high sense of self-efficacy in using AI can more effectively direct the robots to complete these tasks, saving significant time and physical effort, thereby reducing the intensity and stress of their work (10). With a lighter workload, caregivers will have more energy to focus on the individual needs of the older adults and can also reduce the negative emotions caused by work-related fatigue, ultimately improving job satisfaction. Therefore, we propose the following hypothesis.

*H3:* AI self-efficacy has a significant positive impact on the usage satisfaction of caregivers for older adults with care robots.

Furthermore, Yuan found that when older adult caregivers use robots to care for patients with Alzheimer’s disease, those with high AI self-efficacy are better able to master the various functions of care robots, making the process of using them smoother and thereby increasing their usage satisfaction with the robots (58). In the context of older adults care institutions, when caregivers for older adults feel confident in their ability to operate care robots, they are likely to approach the technology with a more open mindset and less anxiety (59), enabling them to fully explore the robots’ capabilities. As a result, caregivers for older adults can experience the tangible benefits the robots provide, leading to greater satisfaction with their use. Therefore, the present study posits that caregivers’ AI self-efficacy in older adults care institutions will significantly influence their usage satisfaction with care robots. Based on the above theoretical and empirical foundations, the following hypothesis is proposed.

*H4:* AI self-efficacy has a significant positive impact on the job satisfaction of caregivers for older adults.

### 2.4 Job satisfaction and usage satisfaction

Satisfaction is a fundamental concept in organizational behavior that has garnered substantial scholarly attention (60). Within older adults care institutions, caregivers regularly engage in diverse caregiving activities to support older adults. The integration of information systems and intelligent technologies into daily operations has been shown to significantly influence users’ job satisfaction (45).

As a convergence of information systems, intelligent technology, and healthcare, care robots are not only poised to become a key tool for addressing pain points in clinical nursing and building a new ecosystem for smart nursing, but they also offer practical, actionable solutions for the intelligent transformation of healthcare and nursing (61). Job satisfaction reflects an individual’s overall affective evaluation, positive or negative, of their work, which in turn shapes their behavioral intentions and actions within professional settings (62).

In the context of older adults care, caregivers’ job satisfaction not only influences their attitudes and behaviors toward caregiving tasks but also affects their usage satisfaction with care robots. Empirical evidence supports this relationship. El-Gazar’s research found that the integration of robots into nursing practice helps boost nurses’ enthusiasm and improve their job satisfaction, which in turn positively influences their satisfaction with the use of robots (63). Similarly, improvements in physicians’ job satisfaction following the adoption of information systems have been linked to increased satisfaction with hospital information system usage (64).

By analogy, it can be inferred that the job satisfaction of caregivers for older adults will influence their satisfaction with the technological tools they are required to use. Therefore, this research puts forward the following hypothesis.

*H5:* Job satisfaction has a significant positive effect on the usage satisfaction of caregivers for older adults with care robots.

**Figure 1:**
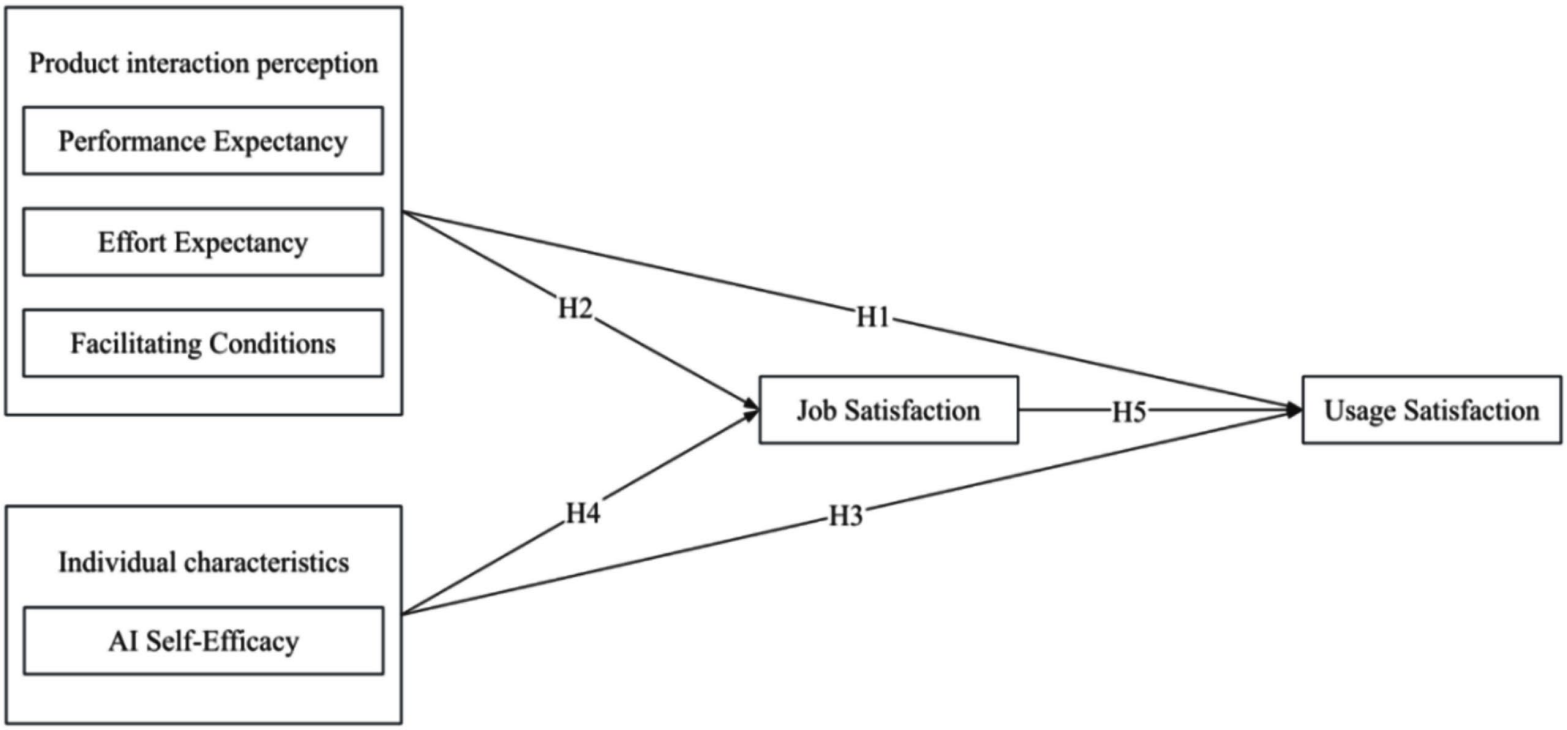
Theoretical model diagram.

Building upon the UTAUT, this research incorporates job satisfaction as a key variable to examine the satisfaction of caregivers for older adults with care robots in older adults care institutions. Additionally, AI self-efficacy is introduced into the model to construct a comprehensive mechanism explaining the factors influencing the usage satisfaction of caregivers for older adults with care robots. Figure 1 illustrates the theoretical model.

## 3 Methodology

### 3.1 Data collection

Offline questionnaire distribution was carried out by the research team from June 2025 to September 2025 in multiple communities and older adult care facilities in Hefei, which is one of the pilot cities for China’s application of AI in senior care and where care robots enjoy high popularity. Each participant took approximately 15–20 min to complete the questionnaire. A total of 600 questionnaires were collected, of which 544 were valid, resulting in a valid response rate of approximately 90.67% (544/600). Details of the data collection are presented in Table 1.

**Table 1:** Sample characteristics (*N* = 544).

Demographic variables	Category	Frequency	Percent (%)
Gender	Male	252	46.3
	Female	292	53.7
Age (years)	18–30	152	27.94
	31–40	162	29.78
	41–50	136	25.00
	≥51	94	17.28
Education level	Junior high school and below	153	28.13
	Technical secondary school/high school	178	32.72
	Junior college	126	23.16
	Bachelor’s or above	87	15.99
Working years	<1	92	16.91
	1–2	136	25.00
	2–3	157	28.86
	≥3	159	29.23
Monthly personal income (RMB)	≤4,000	73	13.42
	4,001–5,000	89	16.36
	5,001–6,000	209	38.42
	≥6,001	173	31.80

### 3.2 Analysis of common method bias

To effectively mitigate potential common method bias, the questionnaire emphasized anonymity to encourage respondents to provide genuine responses and reduce response bias. Second, the data underwent Harman’s single-factor test. The results showed that the first factor explained 42.23% of the variance, which is below the 50% threshold, indicating no severe common method bias in the data (65). Thus, the data demonstrate acceptable reliability and validity.

Furthermore, the Variance Inflation Factor (VIF) values in this study ranged from 1.298 to 2.380, all below 3 (66). This indicates no severe multicollinearity in the data. Therefore, the data are suitable for subsequent analysis in this study.

### 3.3 Measurement

The questionnaire in this study was composed of two parts. In the first part, demographic information of the participants was measured. The second part contained items designed to measure the six constructs included in the proposed model.

All measurement items were adapted from previously published studies. Moreover, prior research was used as a reference for adjusting or revising the measurement questions for each construct. The questionnaire items were measured using a 7-point Likert scale, ranging from 1 (“strongly disagree”) to 7 (“strongly agree”). Table 2 presents the constructs and their corresponding items.

**Table 2:** Constructs and items included in the questionnaire.

Constructs	Items	Measurements	References
Performance expectancy (PE)	PE1	I find that care robots are very useful for my work.	Tomi N. (32, 78, 79)
	PE2	Using care robots enables me to complete tasks more quickly.	
	PE3	I believe that the integration of care robots into my workflow has the potential to significantly enhance my overall work performance.	
	PE4	Using care robots will enhance the efficiency of my work in providing older adults care services.	
Effort expectancy (EE)	EE1	The interaction interface of care robots is clear and easy to understand.	Patil H. (33, 80, 81)
	EE2	I can easily become proficient in using care robots	
	EE3	I believe that care robots are very easy to operate.	
	EE4	It would be easy for me to become skillful at using care robots to provide services for older adults.	
Facilitating conditions (FC)	FC1	I have the necessary resources to use care robots.	Timur Y. P. (82–84)
	FC2	I possess the essential knowledge required for the operation of care robots.	
	FC3	I believe that utilizing care robots is highly compatible with my current work approach.	
	FC4	I can get help from others when I have difficulties in using care robots.	
AI self-efficacy (AI-SE)	AI-SE1	I am confident in my ability to use the tools of care robots appropriately in my work.	Hong J. W. (85–88)
	AI-SE2	I can effectively utilize care robots and perform my work excellently even in challenging situations.	
	AI-SE3	I can apply the skills required for my work through the use of caregiving robots.	
	AI-SE4	Even without the assistance of those around me, I know how to use the nursing robot.	
Job satisfaction (JS)	JS1	I am satisfied with my working environment.	Zhang H. (89–91)
	JS2	I consider my salary and benefits package to be equitable and aligned with industry standards.	
	JS3	I think my working hours are reasonable.	
	JS4	I feel that I am able to coexist harmoniously with my colleagues, the older adults, as well as those in leadership positions.	
Usage satisfaction (US)	US1	Overall, I am satisfied with my experience using the care robots.	Amin M. A. (92–94)
	US2	The functionalities of the care robots effectively align with my operational requirements.	
	US3	I am satisfied with the overall performance and behavior of the care robots.	
	US4	The care robot efficiently fulfilled my need to complete tasks.	
	US5	The nursing robot meets my expectations.	

## 4 Data analysis and results

### 4.1 Data analysis

This study uses SPSS (version 27; IBM Corp. 2020) for descriptive statistical analysis. The research hypotheses were tested using the partial least squares structural equation modeling (PLS-SEM) method in SmartPLS 4.0 software, which can evaluate both the measurement model and the structural model.

### 4.2 Measurement model

The measurement model was evaluated by analyzing the reliability, convergent validity, and discriminant validity of the constructs. To ensure the reliability of a construct, both the Cronbach’s alpha value and the composite reliability (CR) value need to be 0.70 or above, as suggested by Hair et al. and Kaya et al. (67, 68).

Table 3 indicates that, in this study, Cronbach’s alpha values varied between 0.745 and 0.865, and the CR values ranged from 0.839 to 0.908. All these values exceeded the recommended thresholds.

**Table 3:** Results of convergent validity and reliability.

Constructs	Items	Factor loading	Cronbach’s alpha	CR	AVE
PE	PE1	0.802	0.847	0.897	0.686
	PE2	0.833			
	PE3	0.834			
	PE4	0.844			
EE	EE1	0.723	0.745	0.839	0.567
	EE2	0.815			
	EE3	0.729			
	EE4	0.742			
FC	FC1	0.777	0.766	0.851	0.589
	FC2	0.811			
	FC3	0.764			
	FC4	0.714			
AI-SE	AI-SE1	0.834	0.865	0.908	0.711
	AI-SE2	0.829			
	AI-SE3	0.837			
	AI-SE4	0.873			
JS	JS1	0.811	0.836	0.891	0.671
	JS2	0.866			
	JS3	0.824			
	JS4	0.774			
US	US1	0.77	0.831	0.881	0.597
	US2	0.804			
	US3	0.759			
	US4	0.791			
	US5	0.737			

PE, performance expectancy; EE, effort expectancy; FC, facilitating conditions; AI-SE, AI self-efficacy; JS, job satisfaction; US, usage satisfaction; CR, composite reliability; AVE, average variance extracted.

Moreover, as shown in Table 3, all constructs had factor loading values that exceeded 0.7. There is a strong correlation between the observed variables and their corresponding constructs, guaranteeing the reliability of the assessed measures (69). Each construct had an average variance extracted (AVE) above 0.5, indicating that the observed variables adequately explained the corresponding construct dimensions.

To examine discriminant validity, the correlations between constructs were compared with the square roots of the AVE (70). Traditional discriminant validity is assessed using two criteria: the Fornell–Larcker method and cross-loadings. However, both approaches tend to overestimate factor loadings and underestimate relationships between variables, leading to easy passage of discriminant validity tests (71).

The Heterotrait–Monotrait ratio (HTMT) represents a superior method for assessing discriminant validity. If the HTMT value between any two variables is less than 0.85, the questionnaire is considered to possess good discriminant validity. The results show that all HTMT values are below 0.85 (72). Therefore, this questionnaire demonstrates strong discriminant validity (Table 4).

**Table 4:** Distinct validity analysis (HTMT).

Variable	AI-SE	EE	FC	JS	PE	US
AI-SE						
EE	0.805					
FC	0.7	0.714				
JS	0.709	0.608	0.693			
PE	0.752	0.734	0.824	0.701		
US	0.778	0.709	0.781	0.825	0.844	

PE, performance expectancy; EE, effort expectancy; FC, facilitating conditions; AI-SE, AI self-efficacy; JS, job satisfaction; US, usage satisfaction; HTMT, Heterotrait–Monotrait ratio.

### 4.3 Structural model

The structural model, which is the second stage of the PLS-SEM analysis, is used to examine causal relationships between variables. Additionally, direct effects are reflected by path coefficients, as mentioned by Dah and Paul and Manley et al. (73, 74).

The bootstrapping method (5,000 subsamples) was used to test the proposed hypotheses and path coefficients. Table 5 shows that hypotheses H1a, H1c, H2a, H2c, H3, H4, and H5 were supported, whereas H1b and H2b were not supported.

**Table 5:** Hypothesis testing results.

Hypothesis	*β*	*t*-value	*p*-value	Result
H1a	0.290	7.297	0.000	Supported
H1b	0.056	1.570	0.117	Not supported
H1c	0.125	4.270	0.000	Supported
H2a	0.232	3.517	0.000	Supported
H2b	0.029	0.610	0.542	Not supported
H2c	0.206	3.819	0.000	Supported
H3	0.171	5.559	0.000	Supported
H4	0.317	4.834	0.000	Supported
H5	0.328	7.965	0.000	Supported

*β*, standardized path coefficient.

First, the positive effects of performance expectancy (*β* = 0.290, *p* < 0.001) and facilitating conditions (*β* = 0.125, *p* < 0.001) on usage satisfaction are confirmed, supporting H1a and H1c. However, the effect of effort expectancy (*β* = 0.056, *p* > 0.05) on usage satisfaction was not significant, and H1b was not supported.

Second, significant positive effects of performance expectancy (*β* = 0.232, *p* < 0.001) and facilitating conditions (*β* = 0.206, *p* < 0.001) on job satisfaction were observed, supporting H2a and H2c. However, the effect of effort expectancy (*β* = 0.029, *p* > 0.05) on job satisfaction was not significant, and H2b was not supported.

Third, a significant positive effect of AI self-efficacy (*β* = 0.171, *p* < 0.001) on usage satisfaction was observed, supporting H3. In addition, AI self-efficacy (*β* = 0.317, *p* < 0.001) had a significant positive effect on job satisfaction, supporting H4.

Finally, job satisfaction significantly influenced usage satisfaction (*β* = 0.328, *p* < 0.001), supporting H5.

## 5 Discussion

### 5.1 Major findings

This study, grounded in the UTAUT, integrates the construct of job satisfaction and introduces AI self-efficacy as a variable into the theoretical framework to explore the factors influencing the usage satisfaction of care robots among caregivers for older adults in older adults care institutions. The major findings are as follows:

(1) Caregivers’ product interaction perception of care robot usage exerts a partial effect on their usage satisfaction. Specifically, performance expectancy and facilitating conditions significantly enhance usage satisfaction. In contrast, effort expectancy does not demonstrate a statistically significant impact on usage satisfaction. This may be attributed to the relatively low educational attainment of many caregivers for older adults, which could lead to difficulties in operating care robots, thereby attenuating their usage satisfaction (75).
(2) Performance expectancy and facilitating conditions exert a significant positive influence on job satisfaction. However, in this study, effort expectancy does not have a significant impact on job satisfaction. This may be attributed to care robots automatically generating data reports, with management increasingly relying on these “hard metrics” to replace process-based evaluations of caregivers (76). Effort is no longer “seen” by superiors or residents but recorded by sensors, decoupling “working harder” from “receiving positive evaluations.” Consequently, caregivers’ effort expectancy does not significantly influence job satisfaction.

On the other hand, due to a long-term shortage of staff, frequent night shifts, and low social recognition among older adult care workers (77), their “efforts” often appear ineffective in practice. Even when individuals strive to improve service quality, fragmented schedules and physical and mental exhaustion make sustained improvement difficult, thereby eroding confidence in the causal chain linking “effort” to “improvement.”

(3) Both older adults caregivers’ product interaction perception of care robot usage and their levels of AI self-efficacy have a significant positive influence on job satisfaction. When caregivers for older adults hold favorable perceptions of care robots, these technologies can optimize work processes, reduce workload, and ultimately improve work efficiency and service quality. Similarly, higher levels of AI self-efficacy enhance confidence and foster a more positive attitude toward robot adoption, which in turn improves job satisfaction over time. Thus, from both the perspectives of product interaction perception and individual characteristics, these factors significantly affect job satisfaction. These findings indicate that the integration of care robots into daily older adults care services effectively improves working conditions, alleviates work stress, and enhances overall job satisfaction among caregivers for older adults.
(4) AI self-efficacy and job satisfaction among caregivers for older adults have a significant positive influence on their usage satisfaction. Higher levels of AI self-efficacy encourage more proactive engagement with robotic systems in caregiving tasks, fostering habituation and enjoyment, which in turn increases usage satisfaction. Similarly, greater job satisfaction is positively correlated with higher usage satisfaction.

### 5.2 Theoretical implications

There are three aspects to the theoretical implications of this research.

(1) This research proposes and empirically validates a theoretical framework for explaining caregivers’ satisfaction towards care robots. By extending the UTAUT framework, the study identifies key determinants of caregiver’ usage satisfaction. This enriches the theoretical foundation of care robot acceptance research and provides a reference for future scholarly investigations.
(2) This study extends the UTAUT framework by incorporating product interaction perception variables, exploring how caregivers’ perceptions of robotic tools influence both their job satisfaction and care robot usage satisfaction. While prior UTAUT-based studies have predominantly focused on information systems or online platforms, this research applies the theory to the context of care robots in older adults care institutions. This expands the scope and applicability of UTAUT, offering valuable theoretical insights into its utility in emerging technological domains.
(3) By positioning job satisfaction as an endogenous variable within the model, this study examines how individual differences in AI self-efficacy affect the job satisfaction of caregivers for older adults, and subsequently their usage satisfaction with care robots. This advances the theoretical understanding of the interrelationships between job satisfaction and AI self-efficacy, making a meaningful contribution to academic discourse in these fields.

### 5.3 Practical implications

This research additionally holds substantial implications for practitioners.

(1) For caregivers for older adults, this study can effectively identify their actual needs for care robots, optimize the functional positioning of these robots, and thereby improve their job satisfaction. By clarifying the major factors affecting usage satisfaction, it provides a basis for tailoring care robots to better match the practical needs of caregivers for older adults, reducing operational barriers and enhancing their willingness to use such technologies.
(2) For older adults care institutions, exploring the mechanisms underlyingthe usage satisfaction of caregivers for older adults with care robots can facilitate the improvement of intelligent older adults care service systems. This, in turn, can alleviate the care pressure faced by institutions, optimize resource allocation, and enhance their core competitiveness in the industry. Insights from this study can guide institutions in formulating more effective strategies regarding the introduction and application of care robots, ensuring that technological integration aligns with operational needs.
(3) For the government, the findings contribute to evidence-based decision-making regarding the promotion and popularization of AI-enabled older adult care service products. By revealing the practical effects and application conditions of care robots, it provides valuable references for formulating policies related to the development of intelligent older adults care industries, promoting the healthy and sustainable advancement of older adults care service system.

### 5.4 Limitations and future studies

Our study has several limitations that can serve as starting points for future research. First, the data were collected via a cross-sectional survey. As a result, the findings do not capture changes in the usage satisfaction of care robots among caregivers for older adults over time. Future research may adopt longitudinal designs combined with qualitative or empirical approaches to explore how usage satisfaction evolves over time.

Second, while the UTAUT theory provides a useful structure for apprehending usage satisfaction, it may oversimplify the complex processes underlying the usage of care robots. Future studies could explore more comprehensive models that incorporate additional variables influencing usage satisfaction.

### 5.5 Conclusion

The purpose of this study is to investigate the satisfaction of caregivers for older adults with the use of care robots. The findings provide additional evidence that product interaction perception and AI self-efficacy are associated with usage satisfaction among caregivers for older adults. The results show that product interaction perception and AI self-efficacy have a significant influence on usage satisfaction with care robots. Additionally, caregivers for older adults with higher job satisfaction exhibit greater usage satisfaction.

## Data Availability

The raw data supporting the conclusions of this article will be made available by the authors without undue reservation.
